# Supplementary material for: Counterbalance Economics (CBE): Harnessing Aggregate Markups to Finance Voucher-Based Inequality Reduction
Source: F1000Res. 2026 Jun 15;14:980. Originally published 2025 Sep 24. [Version 3] doi: 10.12688/f1000research.166262.3 (PMC13276486; doi:10.12688/f1000research.166262.3)
Supplement: Peer review history

# Reviewer report: Reviewer response for version 3

**DOI:** 10.5256/f1000research.203154.r493849

**Published:** 2026-06-17

**Authors:** D. Tripati Rao, Praveen Kumar

**Affiliations:** 1 EBE Area, Indian Institute of Management Lucknow, Lucknow, Uttar Pradesh, India; 2 Indian Institute of Management Lucknow, Lucknow, Uttar Pradesh, India

Overall, the authors have reasonably and satisfactorily addressed our concerns - reframing around CBE-adjacent analogues, cautious, restrained (conditional) and nuanced (hedged) causal claims, and the expanded macroeconomic and policy implications discussion reads well.

 The present version may be accepted.

Is the work clearly and accurately presented and does it cite the current literature?

Partly

If applicable, is the statistical analysis and its interpretation appropriate?

No

Are all the source data underlying the results available to ensure full reproducibility?

No source data required

Is the study design appropriate and is the work technically sound?

Partly

Are the conclusions drawn adequately supported by the results?

Partly

Are sufficient details of methods and analysis provided to allow replication by others?

No

Reviewer Expertise:

Applied Monetary-Macroeconomics, Labour Economics and International Economics

We confirm that we have read this submission and believe that we have an appropriate level of expertise to confirm that it is of an acceptable scientific standard.

# Reviewer report: Reviewer response for version 2

**DOI:** 10.5256/f1000research.196036.r470829

**Published:** 2026-05-26

**Authors:** D. Tripati Rao, Praveen Kumar

**Affiliations:** 1 Indian Institute of Management Lucknow, Lucknow, Uttar Pradesh, India; 2 EBE Area, Indian Institute of Management Lucknow, Lucknow, Uttar Pradesh, India

**Peer Review: Counterbalance Economics (CBE)**

**Overall Assessment**

 This is a very interesting paper and explores in a current and thought-provoking issue: whether markup rents can be recouped to compensate for the inequity created by them, but methodological flaws cast doubt on the empirical conclusions.

**Critical Methodological Concerns**

**Sample-treatment mismatch (potentially fatal):** Four countries have CBE codes: Germany (2022); France (2022); Sweden (2023); and Brazil (2023) implement their policies after the end of the sample in 2016. Only three countries (China, Malaysia, Australia) have been treated and thus can be counted on for identification.

**Construct validity:** CBE dummy measures windfall taxes on extractive sectors imposed by government, not the voucher system that is built on the firm's markup in the theory of the CBE. Thus, the empirical results pertain to the taxation of resources, rather than to the CBE as designed.

**Missed outcome data:** There are 200 Gini observations; 420 observations are missing (~52%). The method of interpolation focused on early years and emerging economies isn't benign to a DiD design that relies on pre-treatment trend behaviour.

**Data error:** Table 2 shows the minimum GDP per capita as 0.00 which is impossible and indicates that there may have been a coding mistake in the estimation process, using a log-transformation.

**Macro and Policy concerns.**

**Ignore inflationary transmission:** A massive increase in the volume of purchasing power provided to low-income groups – the group with the most elastic marginal propensity to consume – generates an actual demand stimulus that may drive up prices of non-tradeable goods and services. The paper assumes that idle capacity absorbs demand without the price effect, which is an assumption that needs to be substantiated through empirical evidence.

**The political economy of the choices of firms to become compliant:** Firms with more political influence and lobbying capability are also those with the highest markups. The paper does not comment on the literature on regulatory capture, or the reasons why high-markup firms might agree to a rule-based commitment to forgo their rents directly, without regulatory arbitrage or exit.

 A paper's main claim is that it has strong potential in emerging economies, but it is precisely these contexts in which the majority of low-income workers and small businesses are outside of any formal system of mark-ups reporting or system of accepting vouchers. The paper underestimates the institutional conditions that would have to be put in place to scale CBE here.

**No long-run growth accounting:** Redistribution through markup rents can decrease the return to innovation if markups partially offset recovery of the fixed costs and R&D. The paper neither models nor tests for long-run viability of a policy, which is necessary for a policy proposal to be viable over time.

**Interacting with the current tax-transfer systems:** CBE will be working alongside progressive income taxes, VAT, social insurance, and means-tested transfers. The paper does not provide any analysis of how these systems play out, either in terms of complementarity (CBE could be additive) or substitution (CBE could displace political support for a better universal system) or whether there is a mutual crowding out.

Is the work clearly and accurately presented and does it cite the current literature?

Partly

If applicable, is the statistical analysis and its interpretation appropriate?

No

Are all the source data underlying the results available to ensure full reproducibility?

No source data required

Is the study design appropriate and is the work technically sound?

Partly

Are the conclusions drawn adequately supported by the results?

Partly

Are sufficient details of methods and analysis provided to allow replication by others?

No

Reviewer Expertise:

Applied Monetary-Macroeconomics, Labour Economics and International Economics

We confirm that we have read this submission and believe that we have an appropriate level of expertise to confirm that it is of an acceptable scientific standard, however we have significant reservations, as outlined above.

# Response

**Published:** 2026-05-27

**Authors:** William Pacheco

**Affiliations:** Economics, University Anahuac Mexico, Huixquilucan de Degollado, State of Mexico, Mexico

We sincerely thank Professors Rao and Kumar for their careful reading of our manuscript and for their constructive and detailed comments. We recognize the importance of the methodological, conceptual, and policy concerns raised, particularly regarding treatment coding, construct validity, missing data, empirical identification, and the broader institutional and macroeconomic implications of the CBE framework. We will carefully address these issues in a revised version of the manuscript and provide a detailed point-by-point response alongside the revision.

# Reviewer report: Reviewer response for version 2

**DOI:** 10.5256/f1000research.196036.r455667

**Published:** 2026-02-27

**Authors:** Marina da Silva Sanches

**Affiliations:** 1 University of São Paulo, São Paulo, Brazil

The authors have satisfactorily addressed my previous comments.

Is the work clearly and accurately presented and does it cite the current literature?

Yes

If applicable, is the statistical analysis and its interpretation appropriate?

Yes

Are all the source data underlying the results available to ensure full reproducibility?

Yes

Is the study design appropriate and is the work technically sound?

Yes

Are the conclusions drawn adequately supported by the results?

Partly

Are sufficient details of methods and analysis provided to allow replication by others?

Yes

Reviewer Expertise:

Macroeconomics

I confirm that I have read this submission and believe that I have an appropriate level of expertise to confirm that it is of an acceptable scientific standard.

# Reviewer report: Reviewer response for version 1

**DOI:** 10.5256/f1000research.183226.r421087

**Published:** 2025-10-29

**Authors:** Marina da Silva Sanches

**Affiliations:** 1 University of São Paulo, São Paulo, Brazil

**1. General Assessment**

 The manuscript presents a carefully constructed econometric analysis of the *Counterbalance Economics* (CBE) policy and its potential to mitigate the inequality-enhancing effects of corporate markups. The methodology is robust, including multiple identification strategies (DiD, event-study, Driscoll-Kraay standard errors, placebo tests), subsample checks, and sensitivity analyses. The results are internally consistent, economically meaningful, and aligned with the existing literature on market power and inequality.

**2. Strengths**

- **Methodological rigor:** The empirical analysis is well-designed, following best practices in applied econometrics (Callaway & Sant’Anna, 2021; Angrist & Pischke, 2009).
- **Clarity and detail:** Construction of variables, interaction effects (Markup x CBE), and quantitative interpretation are clearly explained.
- **Relevance:** The study addresses a timely topic, linking inequality, corporate market power, and innovative redistribution mechanisms.
- **Empirical robustness:** Placebo tests, dynamic event studies, and subsample analyses confirm that results are not driven by outliers or spurious correlations.

**3. Key Critiques and Limitations**

1. **Overly optimistic interpretation:** While CBE shows potential, the manuscript often overstates policy implications, implying broad, transformative effects that go beyond what the data support. Statements such as *“usher in an era of capitalism that achieves both prosperity and fairness”* should be tempered to reflect conditional effectiveness.
2. **Institutional and conceptual limitations:** a) CBE depends on corporate participation, voluntary compliance, and market structure, and cannot fully substitute state-led redistribution. b) Drawing an analogy between Brazil’s Bolsa Família and CBE is conceptually fragile. Bolsa Família is financed through **progressive taxation** and embedded in a **state-led redistributive framework**, whereas CBE relies on firms’ markup rents and decentralized exchange. The former operates within a logic of universal public provision and democratic accountability; the latter is market-oriented and voluntary. Any resemblance is superficial-both involve in-kind elements, but institutional architectures, objectives, and normative foundations diverge fundamentally (Fiszbein & Schady, 2009; Soares et al., 2010).
3. **Evidence on universal public services:** Empirical research shows that **state-led, universal public services are more effective** than market-based or voucher schemes in reducing inequality and fostering inclusive growth. Expansion of health, education, and care systems significantly lowers income dispersion and enhances intergenerational mobility. Public service investment additionally supports aggregate demand and long-term productivity through human capital formation and social infrastructure. See, for example, the literature discussion in Verbist et al. 2012 (The Impact of Publicly Provided Services on the Distribution of Resources: Review of New Results and Methods – OECD).
4. **Need for context on redistribution limits:** Welfare states in many countries have historically used taxes and transfers to achieve moderate redistribution; the extent of inequality reduction depends on fiscal capacity, political will, and institutional design. Evidence shows that well-designed programs can substantially reduce inequality, particularly when combined with universal services. See, for example, the Bolsa Família program in Brazil, which the paper discusses and cites relevant literature on.

**4. Recommendations for Improvement**

1. **Adjust the tone:** Temper normative and overly definitive statements in conclusions.
2. **Include institutional discussion:** Clarify that CBE’s effectiveness depends on corporate participation, regulatory oversight, and interaction with existing public policies.
3. **Situate CBE relative to public provision:** Explicitly state that CBE is **complementary**, not a substitute, for universal services and traditional redistributive mechanisms.
4. **Cite additional literature:** Include references on the limits of private redistribution and the effectiveness of public services to contextualize findings.

**5. Overall Assessment**

 The manuscript represents a **valuable empirical contribution**. Its econometric design is rigorous and findings are relevant, but the discussion and conclusions should be revised to align with the evidence and acknowledge practical, institutional, and conceptual limitations. With these improvements, the manuscript would be suitable for indexing.

Is the work clearly and accurately presented and does it cite the current literature?

Yes

If applicable, is the statistical analysis and its interpretation appropriate?

Yes

Are all the source data underlying the results available to ensure full reproducibility?

Yes

Is the study design appropriate and is the work technically sound?

Yes

Are the conclusions drawn adequately supported by the results?

Partly

Are sufficient details of methods and analysis provided to allow replication by others?

Yes

Reviewer Expertise:

Macroeconomics

I confirm that I have read this submission and believe that I have an appropriate level of expertise to confirm that it is of an acceptable scientific standard, however I have significant reservations, as outlined above.

# Response

**Published:** 2026-01-31

**Authors:** William Pacheco

**Affiliations:** Economics, University Anahuac Mexico, Huixquilucan de Degollado, State of Mexico, Mexico

We sincerely thank the reviewer for the careful reading of the manuscript and for the thoughtful, constructive, and technically informed comments. We greatly appreciate the positive assessment of the econometric design, robustness checks, and empirical contribution of the paper. The reviewer’s feedback has been extremely valuable in helping us refine the interpretation, institutional framing, and policy positioning of Counterbalance Economics (CBE).
